# The Impact of Emotional States on Construction Workers’ Recognition Ability of Safety Hazards Based on Social Cognitive Neuroscience

**DOI:** 10.3389/fpsyg.2022.895929

**Published:** 2022-06-16

**Authors:** Dan Chong, Anni Yu, Hao Su, Yue Zhou

**Affiliations:** ^1^Department of Management Science and Engineering, Shanghai University, Shanghai, China; ^2^Shanghai Urban Construction Road Engineering Co., Ltd, Shanghai Road & Bridge (Group) Co., Ltd, Shanghai, China

**Keywords:** safety management, construction workers, emotional states, safety hazards, galvanic skin response

## Abstract

The construction industry is one of the most dangerous industries with grave situation owing to high accident rate and mortality rate, which accompanied with a series of security management issues that need to be tackled urgently. The unsafe behavior of construction workers is a critical reason for the high incidence of safety accidents. Affective Events Theory suggests that individual emotional states interfere with individual decisions and behaviors, which means the individual emotional states can significantly influence construction workers’ unsafe behaviors. As the complexity of the construction site environment and the lack of attention to construction workers’ emotions by managers, serious potential emotional problems were planted, resulting in the inability of construction workers to effectively recognize safety hazards, thus leading to safety accidents. Consequently, the study designs a behavioral experiment with E-prime software based on social cognitive neuroscience theories. Forty construction workers’ galvanic skin response signals were collected by a wearable device (HKR-11C+), and the galvanic skin response data were classified into different emotional states with support vector machine (SVM) algorithm. Variance analysis, correlation analysis and regression analysis were used to analyze the influence of emotional states on construction workers’ recognition ability of safety hazards. The research findings indicate that the SVM algorithm could effectively classify galvanic skin response data. The construct ion workers’ the reaction time to safety hazards and emotional valence were negatively correlated, while the accuracy of safety hazards recognition and the perception level of safety hazard separately had an inverted “U” type relationship with emotional valence. For construction workers with more than 20 years of working experience, work experience could effectively reduce the influence of emotional fluctuations on the accuracy of safety hazards identification. This study contributes to the application of physiological measurement techniques in construction safety management and shed a light on improving the theoretical system of safety management.

## Introduction

With a large number of employees, the construction industry is a typical labor-intensive industry worldwide. Every year, over 60,000 work-related fatalities are reported from construction workplaces around the world (Lingard, 2013). The construction industry is a pillar industry in China, from 2000 to 2020, the number of people in the construction industry has increased from 19.94 million to 53.67 million, with an annual benefit of 729.96 billion RMB in the construction industry in 2020, an increase of 3.5% over the previous year (China National Bureau of Statistics, 2020). However, the occupational safety of construction workers is not guaranteed with frequent safety accidents in the construction industry. The total number of safety accidents in the construction industry has remained high over the years (China Construction Industry Association, 2020). According to the Ministry of Housing and Urban–Rural Development of China, 773 production safety accidents occurred in 2019 in housing and municipal engineering in China, with 904 deaths, an increase of 39 safety accidents and 64 fatalities over 2018, up 5.31 and 7.62%, respectively (Ministry of Housing and Urban–Rural Development of China, 2019). In other countries, construction casualty rates are also disproportionately high compared to the number of people employed (Al-Bayati et al., 2019; Liu et al., 2020). Overall, the construction industry continues to experience a disproportionate share of work-related injuries and illnesses, which significantly contributes to work-related fatalities (Pandit et al., 2019). With the frequent occurrence of safety accidents in the construction industry, the aim of research on safety management has gradually changed from the specific environment to human factors. Currently most of the construction workers in China are migrant workers, who have strong risk-taking and fluke psychology. The coarse management mode in the construction industry fails to take care of the psychological needs of the employees and brings serious hidden mental health problems.

Accident Causation Theory suggests that human factors are the main factor in the frequency of safety accidents (Salminen and Tallberg, 1996). Accurate identification and assessment of the potential consequences of construction site safety hazards by construction workers is an important prerequisite for safety management (Farmer and Chambers, 1929). The better the construction workers’ ability to identify and assess the visible or potential safety hazards in the construction sites, the smaller are the chances of their unsafe behaviors occurring (Perlman et al., 2014; Namian et al., 2016). Through a statistical analysis of the causes of 75,000 injuries and fatalities occurred in enterprises, American safety engineer Heinrich concluded that more than 88% of safety accidents were caused by unsafe human behavior (Heinrich, 1941). The analysis of safety accident surveys in the construction industry also showed that unsafe behavior of construction workers was a common cause of safety accidents (Haslam et al., 2005). The main factors affecting unsafe behavior of construction workers can be classified into three aspects: individual factors, organizational factors and environmental factors (Abdelhamid and Everett, 2000; Zhou et al., 2008). Individual influences include psychological factors, physiological factors and the physical quality of the worker, which lead to unsafe behavior of the worker under a single or multiple factors (Alizadeh et al., 2015; Namian et al., 2016). An individual’s safety behavior is affected by emotional state; Affective Event Theory (AET) suggests that employees’ behavior and performance at work are largely determined by the changes in their emotions at each moment rather than their attitudes or personalities (Weiss and Cropanzano, 1996). The AET has been demonstrated effectively in the areas of mine worker safety behavior (Yang et al., 2020), driver driving safety (Muller et al., 2014) and among other areas. Kajiwara verified that emotions can influence the productivity and accuracy of workers in a logistics picking system (Kajiwara et al., 2019). Manzoor developed an agent-based computational social agent model to explore how decisions can be affected by regulating the emotions involved, and how emotions are affected by emotion regulation and contagion (Manzoor and Treur, 2015). However, people do not always think rationally when they act, thus they often make irrational choices or decisions when they are “emotionally driven.” Therefore, employees’ emotional states and the long-term emotions accumulated from emotional fragments can interfere with individual decisions and behaviors (Rachlin, 2003).

The psychological factors are considered as the most important contributors of unsafe behavior for construction workers, and the psychological activities are influenced by individual emotions. Ekman divided emotions into six basic emotions that are sadness, happiness, anger, disgust, fear and surprise (Ekman, 1992b). Zelenski classified all emotional states into positive and negative emotions (Zelenski et al., 2012). Emotions can be measured in three dimensions including personal physiological changes, subjective feelings and external expressions (Kim et al., 2013). When an emotion occurs, it will increase the heart rate, dopamine secretion or brain activity. These changes can be reflected by physiological signals, such as galvanic skin response, blood pressure, respiration amplitude and brain waves, which can be collected by wearable devices in real time (Dzedzickis et al., 2020). Galvanic skin response is a highly relevant physiological signal for individual emotions. Four-channel biosensors were used to measure electromyogram, electrocardiogram, skin conductivity and respiration changes, by using an extended linear discriminant analysis (pLDA), Kim et al. developed a novel scheme of emotion-specific multilevel dichotomous classification (EMDC) with an accuracy of 95% (Kim and André, 2008). Zhao et al. (2018) used a sensor-enriched wearable wristband to measure the three physiological signals including blood volume pause, electrodermal activity and skin temperature. They classify the emotions into four types in aspect of arousal and valence. Zhang choose four physiological signals including photoplethysmography, galvanic skin response, respiration amplitude and skin temperature. Recursive Feature Elimination-Correlation Bias Reduction-Support Vector Machine (SVM-RFE-CBR) algorithm was used for the classification (Chen et al., 2020). Izard et al. determined an individual’s emotions by questionnaire (Dougherty et al., 1974). Watson proposed the positive and negative emotion scale, ten adjectives were applied to express their individual emotions at work, and the results reflect the individual’s accumulated emotions and emotional experience (Watson et al., 1988). This scale can describe the emotions effectively for its simplicity and interpretation. External expressions refer to the external changes that can be visually observed under a stimulus, such as changes in facial expressions, tone of voice and behavior. Nevertheless, this method lacks objectivity because the external performance of individuals can be hidden or disguised (Ekman, 1992a).

The affective generalization theory (Johnson and Tversky, 1983) suggests that emotions irrelevant to the decision-making task will affect people’s judgments about the probability of events with the same emotion valence. Specifically, positive emotions reduce the subjective estimate of risky events and people in positive mood are prone to perform risky behaviors, while people in negative emotions are prone to perform risky behaviors. In contrast, Mood Maintenance Hypothesis (Isen and Patrick, 1983) is the other classic theory about emotional valence, and it refers to people’s tendency to maintain positive mood states and implies that positive mood is associated with less critical thinking and reduced information processing, and is prone to reduce their estimates of risk events and less likely to take risky behavior. Positive emotions promote brain mental activity and thus, can enable individuals maintain a higher level of concentration (Pool et al., 2016). Fredrickson found the relationship between positive emotions and unsafe behaviors has a U-shaped effect (Fredrickson and Branigan, 2005), while negative emotions can decrease individual’s attention, responsiveness and reasoning abilities (MacLeod et al., 1986). The more intense and emotional the workers are, the more likely they are to commit intentional violations, leading to unsafe accidents (Radenhausen and Anker, 1988; Golparvar, 2016).

Research on emotions suggests that there are diverse effects of emotions on individuals’ behavior. Theoretical controversies over the emotion maintenance hypothesis and the affective generalization theory remain. Given the specificities of construction task and the construction worker population, this paper examines the effects of emotions on construction workers’ recognition of safety hazards from a safety management perspective. A wearable device (HKR-11C+) was used to collect physiological signals for emotion classification, which is more objective compared with subjective questionnaire traditionally used in previous studies. In addition, this study achieves a quantitative analysis between emotions and individual behavior through the quantification of emotional valence. This research is helpful for construction workers to regulate their self-safety behaviors from an individual psychological perspective, as well as provide theoretical safety management strategies that focus on individual psychology for construction companies.

## Materials and Methods

The famous James Lange’s theory divides emotions into two basic dimensions that are emotional arousal and emotional valence (Lang, 1995). Emotional arousal refers to the level of activation of an individual’s emotion in response to a stimulus from passive to active, while emotional valence describes the level of pleasant or unpleasant experience from negative to positive. Emotions in this study were classified into positive, neutral and negative emotions by emotional valence. The construction workers’ recognition ability of safety hazards is measured in three aspects including the reaction time to safety hazards, identification accuracy of safety hazards, and the perception level of safety hazards.

### Participants

Thirty students from Shanghai University majoring in construction engineering management were selected for the pilot test to confirm the feasibility and validity of the experiment. Forty construction workers from six Shanghai construction engineering enterprises were recruited for the formal experiment. Among all the subjects, 3 are construction workers and the other 34 were workers in supervisory positions. There are 3 subjects were below undergraduate level, 27 were undergraduates and 10 were postgraduates. All subjects had received safety management training of construction work. The studies involving human participants were reviewed and approved by Ethics Committee of Shanghai University. Participants selected for the pilot and formal experiment were based on the following criteria: (1) Familiar with the construction industry or have long-term working experience on construction site, and familiar with the operation codes on the construction site; (2) Physically and mentally healthy without any psychological disorders; (3) All are right-handed; (4) All provided written informed consent. The basic information of the subjects are shown in Table 1.

**Table 1:** Basic information of the subjects.

	Category	Number
Gender	Male	37
	Female	0
Years of working experience	1–9 years	12
	10–19 years	13
	20 year above	12
Job type	Operative workers	10
	Supervisory workers	27
Education background	Below undergraduate	6
	Undergraduate	21
	Postgraduate	10

### Procedures

**Figure 1:**
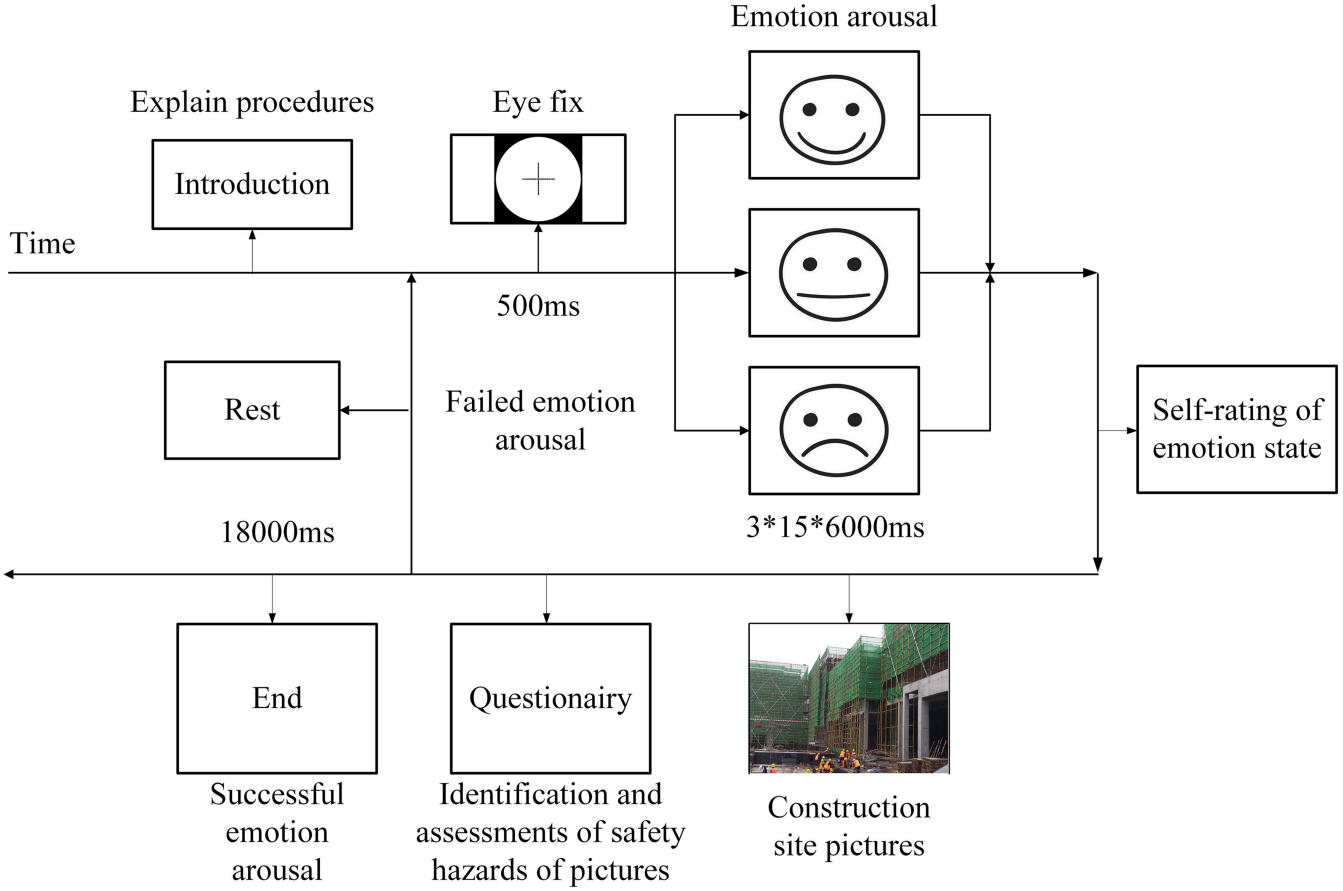
Procedures of the experiment.

The experiment was carried out in a closed construction site conference room without interference. Forty rounds of experiments were included in this study. Each round contains three parts that are positive emotions, neutral emotions, and negative emotions. There are 120 samples in total. After informed consent, all the participants were attached electrodes for the physiological measurement. The experiment guidance is displayed by computer, which explains the purpose and procedures of the experiment. Following the instruction, a picture of the targeted emotion will be shown on the screen to stimulate the empathic effect of the participant, and the picture will last for 6 s, 15 pictures will be shown each time. Subsequently, participants completed the Positive and Negative Affect Schedule (PANAS) (Watson et al., 1988), followed by the identification and assessments of safety hazards in the construction pictures. The experimental procedure is shown in Figure 1.

### Individual Emotional Arousal

Arousal of individual emotions using picture stimuli is one common forms of emotional stimulation (Gerdes et al., 2014). The International Affective Picture System (IAPS) database (Lang et al., 1988) was used to evoke different emotional states of the construction workers. Thirty images each of positive, neutral and negative pictures were selected from the IAPS as emotional arousal stimulus material. Researchers have found that incidental emotions pervasively carry over from one situation to the next, affecting decisions that unrelated to that emotion, known as the carryover of incidental emotion (Loewenstein and Lerner, 2003; Lerner and Tiedens, 2006; Keltner and Lerner, 2010). In the IAPS database, values of valence indicating the level of enjoyment and the values of arousal indicating the level of excitement. The positive pictures selected in this paper include life scenes, animal activity pictures and baby pictures. Neutral pictures include pictures of static objects, abstract artwork and pictures of natural environment. Negative pictures include catastrophic events, violent and brutal scenes and pictures of disabled individuals. Images of the IAPS database cannot be displayed as a result of a confidentiality agreement. The valence value of the negative neutral and positive mood pictures were 1.78, 4.92, 7.83, and the arousal value was 6.36, 3.37, 5.14, respectively. The mean squared deviation of the pictures was less than 2.4, ensuring the validity of the pictures (Zhang et al., 2018). The subjects were randomly shown one set of emotional pictures, and level of emotional arousal was evaluated using the PANAS Emotional Self-Rating Scale; subjects whose emotions were not aroused were excluded from the results of the experiment, the emotional scale is shown in Table 2.

**Table 2:** The emotional state after emotion arousal.

1	2	3	4	5	6	7	8	9
Very Negative		Negative		Neutral		Positive		Very Positive

After the 15 emotion pictures were displayed, the participants will give a self-evaluation of their emotions by filing an emotion scale adapted from PANAS, with a scale ranging from 1 to 9. The higher scores indicate stronger positive emotions, while the lower scores indicate stronger negative emotions. The emotional scale is shown in Table 3.

**Table 3:** Expected frequency and severity of safety hazard.

Severity/Frequency	Very common	Common	Uncommon	Very uncommon
Negligible	0.19	0.04	0.00375	0.000375
Emergency aid	1.13	0.27	0.0226	0.00226
Seek medical advice	3.2	0.77	0.064	0.0064
Hospitalization	6.4	1.53	0.128	0.0128
Permanent disablement or fatality	340.48	81.55	6.81	0.681
No risk	0	0	0	0

### Galvanic Skin Response Measurement

**Figure 2:**
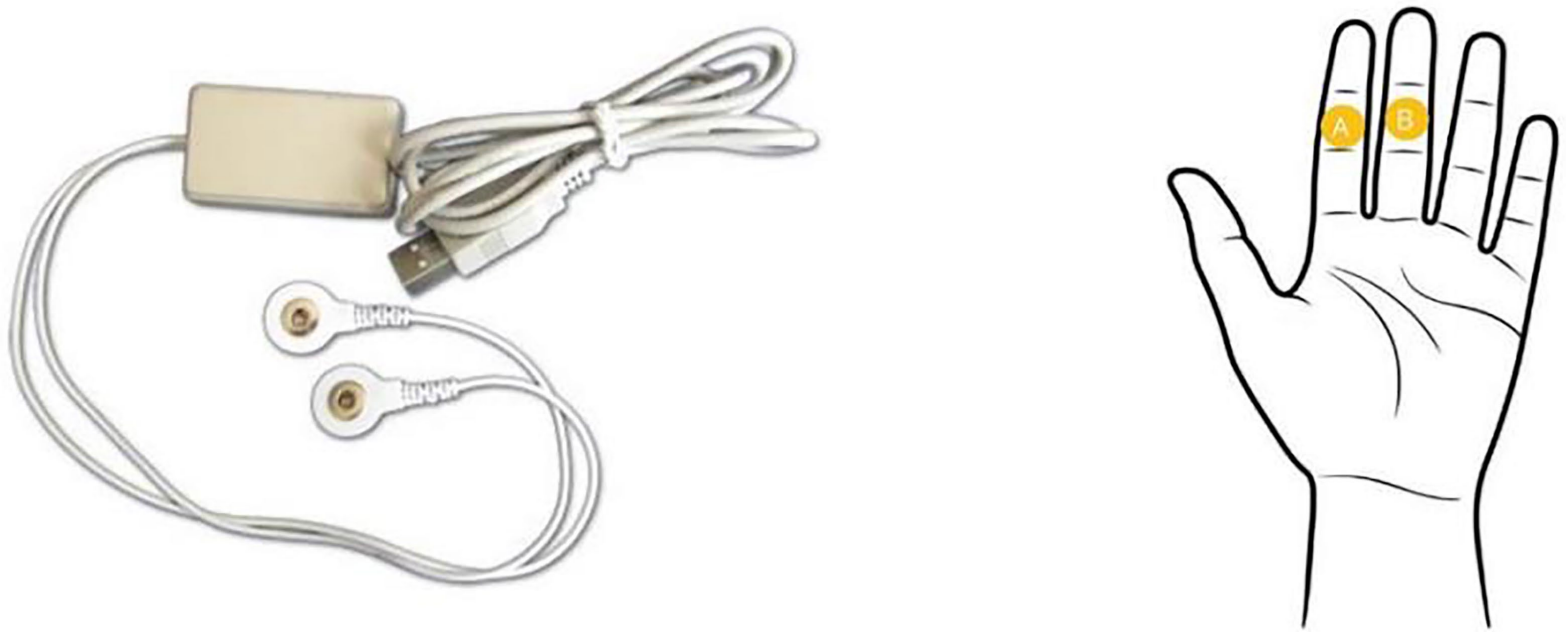
GSR equipment and attachment location.

After cleaning the skin surface, the participants were attached the sensor to the construction worker’s finger. The electrodes were stick in the sensor around the index and middle finger. The galvanic skin response data were recorded and sent to a computer. The picture of the GSR equipment and the electrode attachment location is shown in Figure 2. The galvanic skin response at 5–6 s after the emotional arousal was used for identifying and classifying of the emotions. The experiment was carried out in a laboratory at a room temperature of 22°C, the subjects sitting still in front of a computer for the galvanic skin response measurement, with the temperature and humidity remaining constant throughout the experiment. The signal processing procedures are elaborated in Section 3.

### The Measurement of the Recognition Ability of Safety Hazards

**Figure 3:**
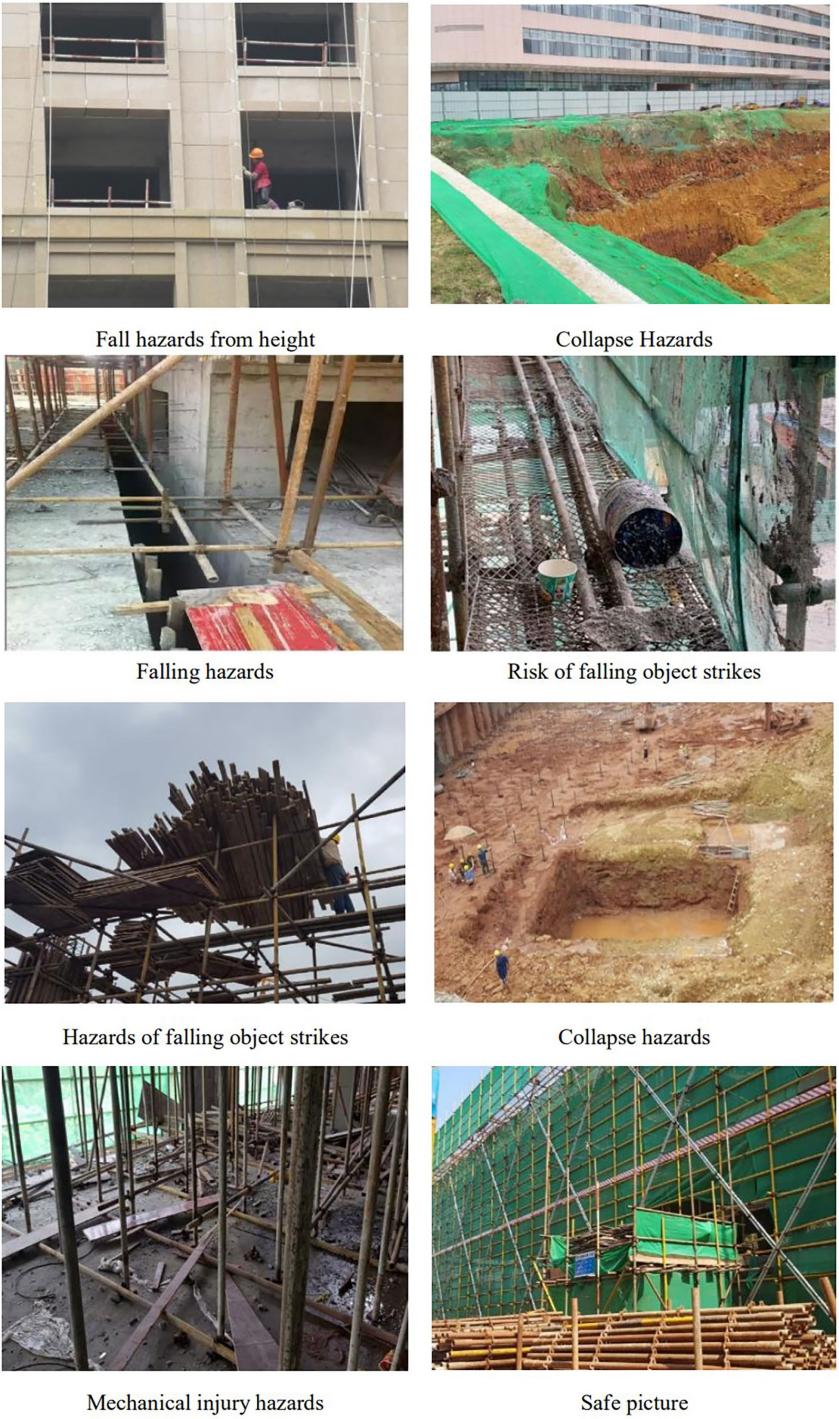
Examples of safety hazards pictures.

The recognition of safety hazards was measured by identifying the safety hazards from construction site pictures, and images of construction sites containing five types of safety hazards were collected for this study from 12 construction sites in Shanghai, China. These images were obtained from safety incident reports, and the opinions of 10 experts were collected to evaluate the two dimensions of the selected images, (1) whether the images visually represented the safety hazards of that type of construction and (2) whether the images were prevalent in the construction site. The 120 images were retained after deleting the lower scoring images. The images were displayed randomly according to category, and 16 images were presented on screen in a set for the subjects to evaluate the safety hazards. The distribution of the 16 images by category was: 7 fall from height, 2 electric shock and fire, 2 object strikes, 1 collapse hazard, 1 mechanical injury and 3 no hazard images. To avoid a learning effect in the subjects, each picture was presented only once at random, with no repeated presentations in all sets for one participants. Some examples of the images chosen are shown in Figure 3.

The cognitive level of safety hazards was measured by behavioral experiment. The participants were requested to view the construction site images and determine if the pictures contain safety hazards (Tixier et al., 2014). The participants pressed “1” on the keyboard if they think there are safety hazards, while “0” on the keyboard if there are not. The computer automatically records the time taken to identify safety hazards and the accuracy of the safety hazards assessment. The perception level of safety hazards of construction workers was measured by safety hazards perception assessment form, which was completed simultaneously when workers believe there is a safety hazards in the given picture. Hallowell pioneered the use of this form by quantifying safety hazards perception as the product of the expected frequency and severity of injury (Hallowell, 2010). The corresponding scores of perception level of safety hazards are shown in Table 3.

### Statistical Analysis

Forty subjects participated in this study, excluding three who failed in emotional arousal with 37 remaining subjects. After 37 sets of experiments, each containing 3 categories of emotional stimuli, 16 pictures of safety hazards for each category, a total of 1776 data were collected. Finally, 1,650 valid data were obtained with a 92.9% validity after removing invalid questionnaires.

The collected data were smoothed and filtered with a median filter and a third-order Butterworth low-pass filter. A cut-off frequency of 0.3 HZ was used to filter out abnormal signals, and the signals were eliminated from the baseline interference to reduce the influence caused by the measurement instrument itself and the current and voltage. The filtered galvanic skin response signals were extracted and normalized from both time domain signal features and descriptive features, and then, principal component analysis is applied to reduce the dimensionality of the acquired features to obtain the signal features for classification.

The reaction time to safety hazards, accuracy of safety hazards identification and perception level of safety hazards of construction workers in different emotional states were analyzed. One-way ANOVA was used to investigate the differences in recognition ability of safety hazards among workers within different age groups under different emotional states. Pearson correlation and regression analyses were used to quantify the effects of emotions on the reaction time to safety hazards, the accuracy of safety hazards identification, and perception level of safety hazards, respectively.

## Results and Discussion

### Classification of Galvanic Skin Response Signals

**Figure 4:**
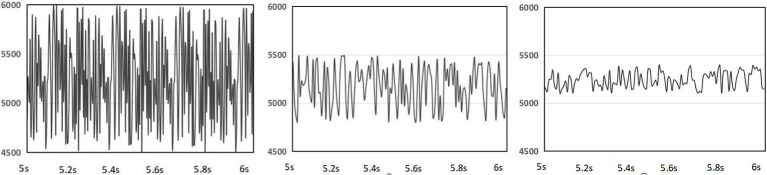
Diagram of the filtering process results.

The collected data were smoothed and filtered with a median filter and a third-order Butterworth low-pass filter, and the cut-off frequency was set as 0.3 HZ to filter out the abnormal signals and retain the valid signals. The original waveform, the waveform after median filtering and the waveform after low-pass filtering are shown in Figure 4; the obtained signals were de-baselined to reduce the effects caused by the measurement instrument itself and the current and voltage; the filtered skin electrical signals were extracted from both time domain signal features and descriptive features. The filtered electrical skin signal was extracted from both time domain signal features and descriptive features, and the extracted features are shown in Table 4. Principal component analysis was used to reduce dimension, the results are shown in Table 5, and the coefficient of the feature GSR_diff_Std is low, so the feature is deleted from the subsequent classification training. The final 10 features retained after normalization were obtained for the subsequent Support Vector Machine classification.

**Table 4:** Selected variables.

No.	Selected Variables	Codename
1	Mean value of GSR	GSR_Mean
2	Standard deviation of GSR	GSR_Std
3	Minimum value of GSR	GSR_Min
4	Maximum value of GSR	GSR_Max
5	First difference mean value of GSR	GSR_diff_Mean
6	First difference standard deviation of GSR	GSR_diff_Std
7	First difference minimum value of GSR	GSR_diff_Min
8	First difference maximum value of the of GSR	GSR_diff_Max
9	Average magnitude of GSR	GSR_AveMag
10	Average rise time of GSR	GSR_AveRt
11	Average energy of GSR	GSR_AveE

**Table 5:** Results of principal component analysis.

	1	2	3	4	5
GSR_diff_Min	0.829	0.452	−0.254	0.196	0.021
GSR_diff_Mean	0.826	0.457	−0.251	0.200	0.014
GSR_diff_Max	0.769	0.526	−0.275	0.223	0.017
GSR_Min	0.767	−0.552	0.293	−0.124	0.069
GSR_Max	0.767	−0.551	0.293	−00.124	0.070
GSR_Mean	0.766	−0.552	0.293	−0.124	0.069
GSR_AveRt	0.084	0.711	0.569	−0.343	−0.074
GSR_AveE	0.060	0.701	0.675	−0.126	−0.162
GSR_AveMag	−0.063	0.087	0.493	0.732	−0.209
GSR_diff_Std	0.152	0.522	−0.251	−0.547	0.090
GSR_Std	−0.243	0.261	0.263	0.197	0.875

**Figure 5:**
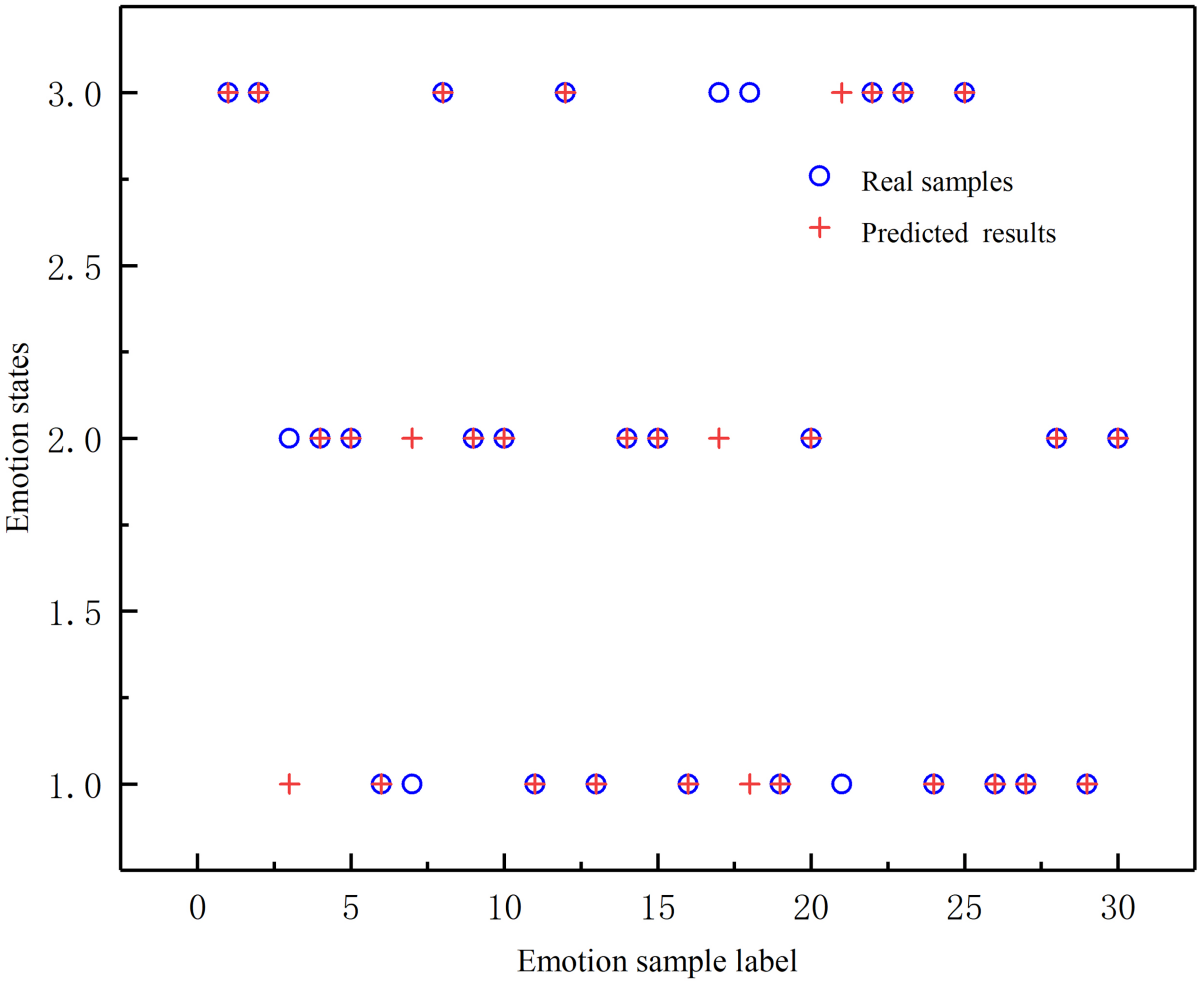
SVM classification prediction result.

The 120 sample points were used as the training data set; the Support Vector Machine (SVM) model was applied for classification training. In a ratio of 2:1, these 120 samples are divided into a training set (90 samples containing 30 positive, 30 neutral, and 30 negative samples) and a test set (30 samples). Both classification accuracy and model validation were improved by classified labelling of these 120 sample points and supervised learning of the model, which was implemented by Matlab R2016b. In the prediction experiment, 30 sample were validated and the results are shown in Figure 5; Table 6. The vertical coordinates 1,2,3 correspond to negative, neutral and positive samples, respectively. When calculating the sensitive, specificity and precision of the examples, for each emotion, the emotion itself is considered a positive example, while the remaining two emotional states are considered negative examples. The classification results indicate that the picture triggering method in this study achieves effective emotional arousal.

**Table 6:** Support vector machine classification simulation training results.

Category	Sensitive	Specificity	Precision	Accuracy	f1
Positive	77.8%	90.5%	70%	–	0.74
Neural	90%	85%	75%	–	0.82
Negative	90.9%	84.2%	76.9%	–	0.83
Integral	–	–	–	86.7%	0.80

### Recognition Ability of Safety Hazards of Construction Workers in Different Emotional States

The statistical results of recognition ability of safety hazards of construction workers in different emotional states are shown in Table 7. Workers in the positive emotional state had the shortest reaction time (5.61 s), and workers in the negative and neutral emotional states required longer reaction time (8.08 s and 6.91 s, respectively). Construction workers in the neutral emotional state had the highest identification accuracy of safety hazards (92.25%) and perception level of safety hazard (24.52), while in the negative emotional state, the construction workers had the lowest identification accuracy of safety hazards (75.41%) and perception level of safety hazards (0.75) than other emotional states.

**Table 7:** Safety hazard cognition results in different emotional states.

Emotional State	Reaction time (s)	Accuracy (%)	Safety hazard perception
Negative	8.08	75.41	0.75
Neural	6.91	92.25	24.52
Positive	5.61	80.60	3.10

The study found that the reaction time for identifying safety hazards was longer in negative emotions than in neutral and positive emotions. When construction workers were in a positive emotion, the feedback time for identifying safety hazards in construction site pictures was 5.61 s, which was less than the 6.91 s in a neutral emotion and 8.08 s in a positive emotion. This conclusion is consistent with Fredrickson’s findings that positive emotions serve to improve individual’s physical, intellectual, and perceptions (Fredrickson, 1998). Although the reaction time to safety hazards became shorter, the accuracy identification of safety hazards decreased when the emotional valence increased from negative to neutral. The identification accuracy of safety hazards was 92.25% under neutral emotional state, which was greater than the negative emotional state (75.41%) and the positive emotional state (80.60%). The findings are similar to the findings on the effect of emotion on driver performance, with drivers performing better in a neutral state (Jeon et al., 2014) hazards.

### The Effect of Emotions on the Recognition Ability of Safety Hazards of Different Working Age Groups

Given that the construction workers’ emotions may be influenced by the age (Kappes et al., 2017), age was chosen as the independent variable and a one-way ANOVA was used to explore the effect of emotions on construction workers’ recognition ability of safety hazards at different working ages.

#### Reaction Time

The results of the one-way ANOVA for the reaction time to safety hazards at different working ages are shown in Table 8. The significant differences between the different age groups indicate that age has a significant effect on the reaction time to safety hazards for construction workers. Correlation-type effect size eta square (Ferguson, 2009) and power reflect that age has a small estimates effect size on the reaction time of construction worker.

**Table 8:** One-way ANOVA results for reaction time at different working ages.

Emotional states	Working years	Mean value	Standard deviation	Minimum	Maximum	*F*	Sig	*η* ^2^	Power	Multiple Comparisons
Negative	0–9	7.74	1.384	4.87	10.50	9.843	0.000	0.127	0.096	1 < 3
	10–19	8.02	1.675	3.35	12.72					
	20 and above	8.50	1.564	5.06	15.50					2 < 3
Neural	0–9	6.35	1.779	1.43	9.75	21.203	0.000	0.136	0.103	1 < 2
	10–19	6.98	1.523	2.85	10.12					1 < 3
	20 and above	7.49	1.617	5.05	18.50					2 < 3
Positive	0–9	4.42	1.931	0.55	8.95	70.670	0.000	0.096	0.076	1 < 2
	10–19	5.71	1.321	1.03	8.47					1 < 3
	20 and above	6.44	1.052	3.60	9.33					2 < 3

1 represents the 0–9 working years group, 2 represents the 10–19 years working group and 3 represents the 20 and above years working group.

**Figure 6:**
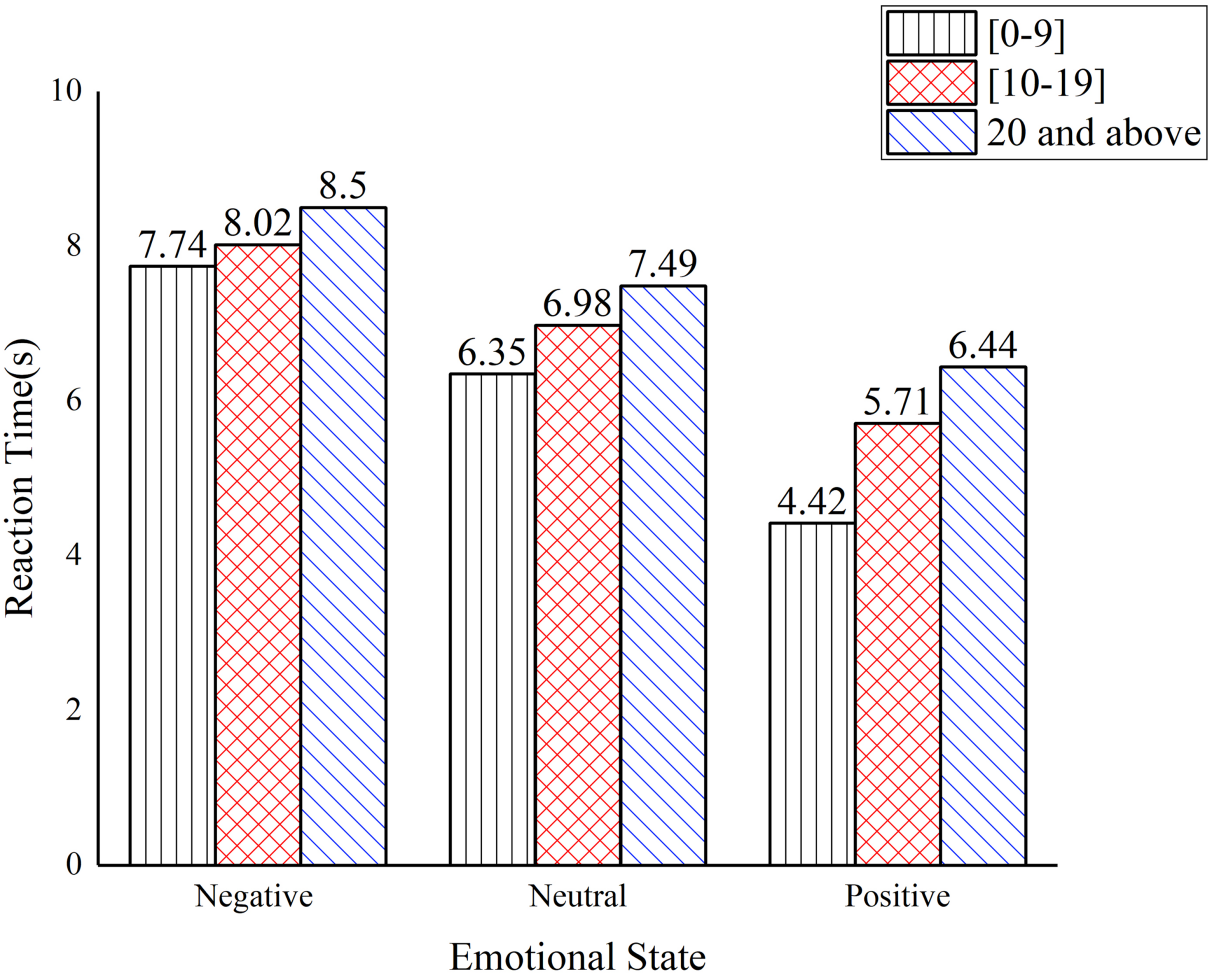
Reaction time to safety hazard under different working ages.

The reaction time to safety hazards under different working ages is shown in Figure 6. In all three emotional states, construction workers with more than 20 years of experience had longest reaction time. Workers with more than 19 years of working experience had longer reaction time than the 10–19 years working experience group by more than 0.5 s, and more than 0.8 s than those with less than 10 years. This suggests that as construction workers increase in years of experience, the reaction time to recognize safety hazards increases, while influence of emotions on the reaction time to safety hazards decreases. This experiment required computer operation and construction workers with more than 20 years of experience, who were generally older and less skilled at operating the devices may have contributed to longer reaction time.

#### Identification Accuracy

The results of the one-way ANOVA for the identification accuracy of safety hazards at different working ages are shown in Table 9. The significant differences between the different age groups indicate that age has a significant effect on the identification accuracy of safety hazards for construction workers. Correlation-type effect size eta square (Ferguson, 2009) and power reflect that age has a small estimates effect size on the identification accuracy of construction worker.

**Table 9:** One-way ANOVA results for identification accuracy at different working ages.

Emotional states	Working years	Mean value	Standard deviation	Minimum	Maximum	*F*	Sig,	*η* ^2^	Power	Multiple Comparisons
Negative	0–9	0.74	0.459	0.510	0.902	4.663	0.010	0.096	0.076	1 < 3
	10–19	0.81	0.446	0.603	0.895					
	20 and above	0.85	0.370	0.537	0.916					
Neural	0–9	0.90	0.242	0.645	0.923	3.978	0.042	0.082	0.069	1 < 3
	10–19	0.94	0.149	0.668	0.952					
	20 and above	0.94	0.236	0.636	0.914					2 < 3
Positive	0–9	0.71	0.439	0.554	0.886	6.166	0.002	0.063		1 < 3
	10–19	0.73	0.395	0.527	0.893				0.061	
	20 and above	0.86	0.351	0.611	0.921					

1 represents the 0–9 working years group, 2 represents the 10–19 years working group and 3 represents the 20 and above years working group.

**Figure 7:**
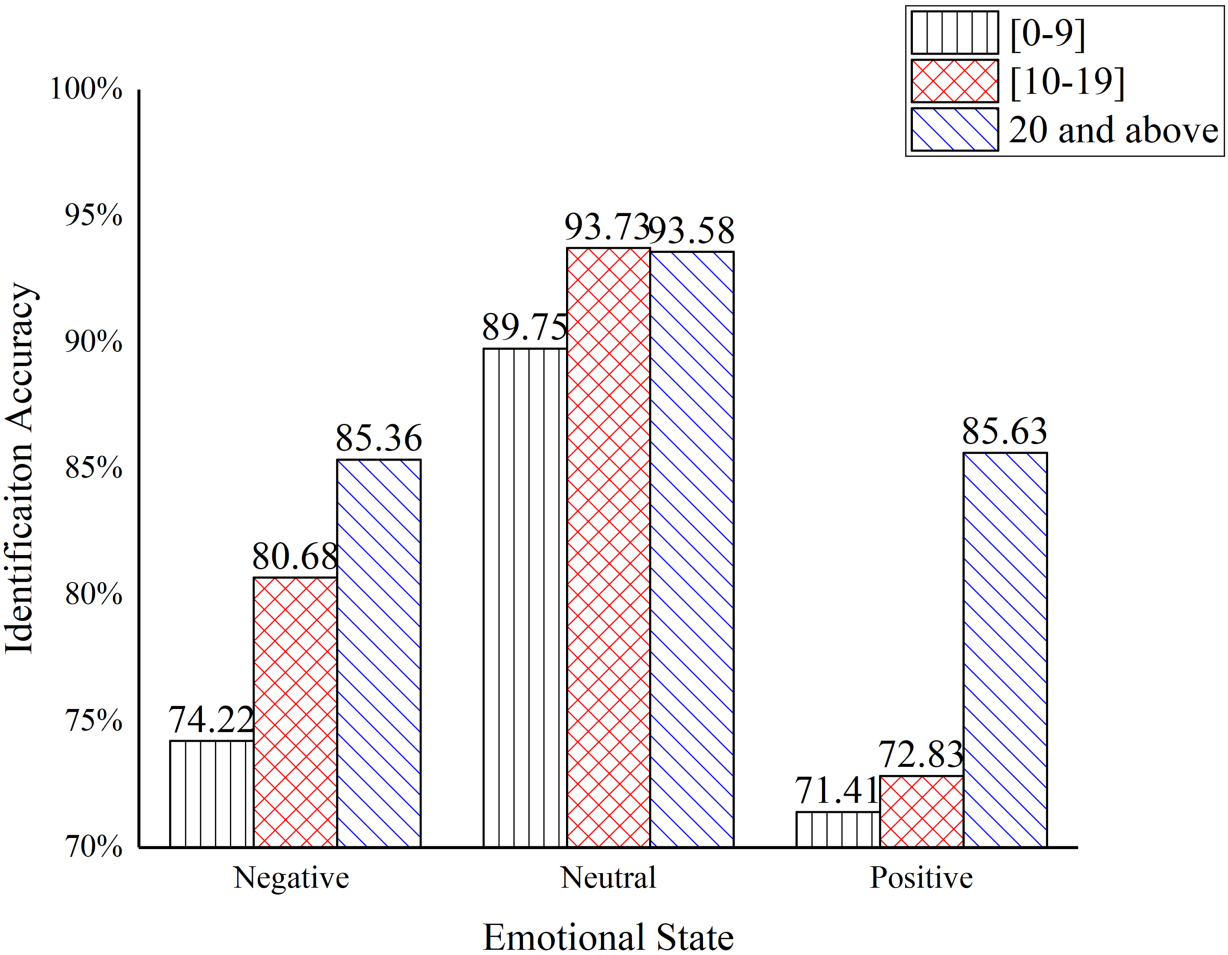
Identification accuracy of safety hazard under different working ages.

The identification accuracy of safety hazards under different working ages is shown in Figure 7. The graph illustrates that the construction workers with more than 20 years of working experience have a higher accuracy in safety hazards identification under positive and negative emotional states than construction workers in the other two age groups. While under neutral emotions, workers with more than 20 years of working experience and the group with 10–19 years have similar accuracy in evaluate safety hazards, at 93.73 and 93.58%, respectively, which are both at a high level. The above results indicate that the accuracy of safety hazards identification of experienced workers with more than 20 years of experience is less affected by their emotional state, which means that work experience can effectively reduce the impact of emotional fluctuations on the accuracy of safety hazards identification.

#### Safety Hazards Perception

The results of the one-way ANOVA for the perception level of safety hazards at different working ages are shown in Table 10. The significant differences between the different age groups indicate that age has a significant effect on the perception level of safety hazards for construction workers. Correlation-type effect size eta square (Ferguson, 2009) and power reflect that age has a small estimates effect size on the safety hazards perception of construction worker.

**Table 10:** One-way ANOVA results for perception level at different working ages.

Emotional states	Working years	Mean value	Standard deviation	Minimum	Maximum	*F*	Sig	*η* ^2^	Power	Multiple Comparisons
Negative	0–9	2.7177	12.7158	0.0037	81.5500	3.004	0.041	0.028	0.052	1 < 2
	10–19	7.1499	20.2831	0.0640	81.5500					
	20 and above	4.1766	16.6419	0.0128	81.5500					
Neural	0–9	13.6066	25.9091	0.1280	81.5500	10.945	0.000	0.023	0.051	1 < 2
	10–19	40.2416	38.2904	0.2700	81.5500			1 < 3	
	20 and above	20.3367	30.7954	0.2700	81.5500			2 > 3	
Positive	0–9	0.57601	1.4384	0.0000	6.8100	33.901	0.000	0.027	0.052	1 < 2
	10–19	6.7472	21.2234	0.0003	81.5500					
	20 and above	1.1089	2.16974	0.0003	6.8100			2 > 3	

1 represents the 0–9 working years group, 2 represents the 10–19 years working group and 3 represents the 20 and above years working group.

**Figure 8:**
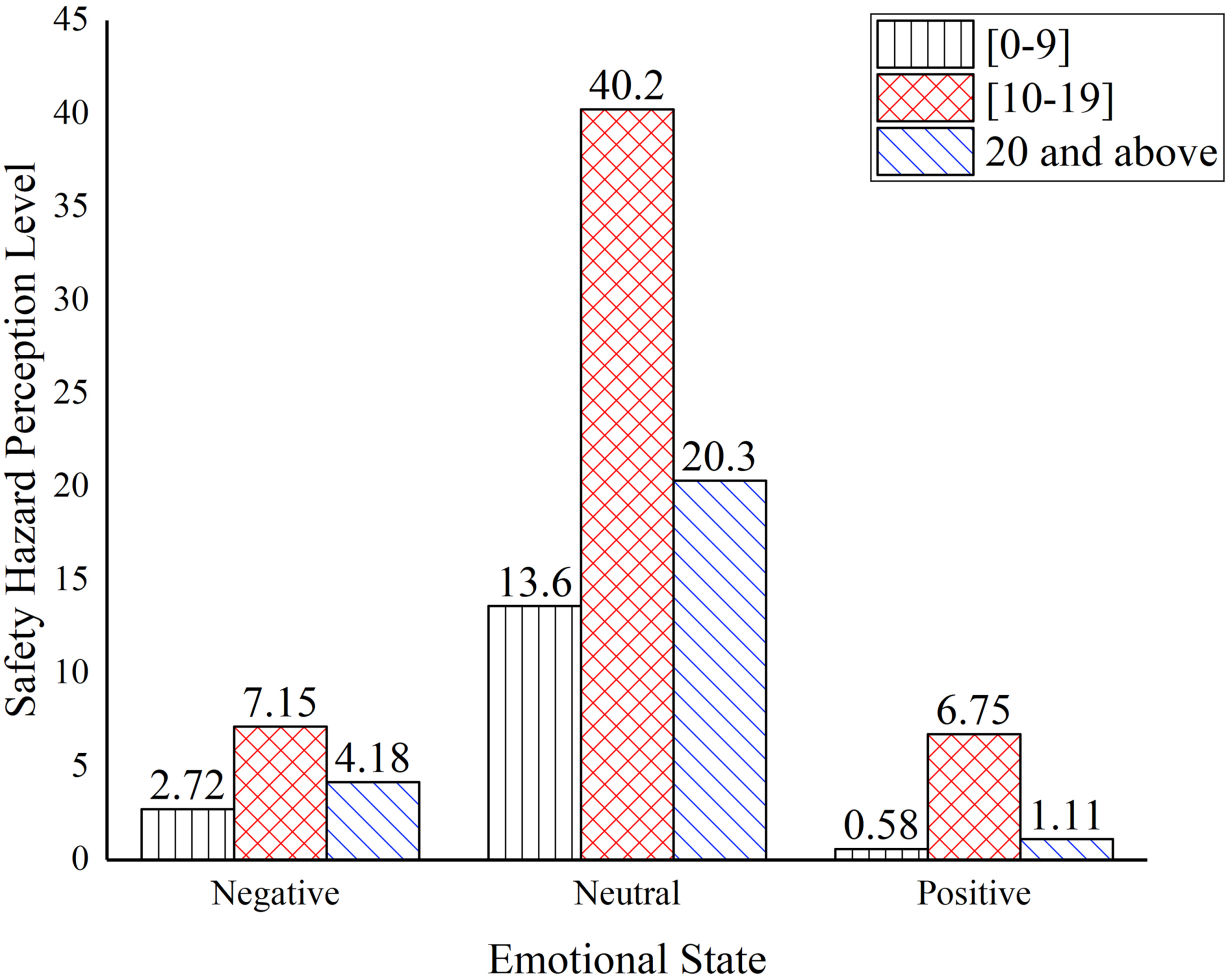
Perception level of safety hazard under different working ages.

The perception level of safety hazards under different working ages is shown in Figure 8. As illustrated in the figure, the highest level of safety hazards perception was found in the group of workers aged 10–19 years old in the neutral mood state, at 40.24, and the group of workers aged 10–19 years old had a higher level of safety hazards recognition in all emotional states. In all age groups, the level of perceived safety hazards increased from low to high then decreased as construction workers changed from extreme negative to extreme positive emotions.

### Quantitative Relationship Between Emotional Valence and Recognition Ability of Safety Hazards

According to the results of safety hazards reaction time, identification accuracy of safety hazards and perception level of safety hazards, correlation and regression analysis were used to explore the quantitative relationship between emotional valence and construction workers’ recognition ability of safety hazards.

#### Emotional Valence and Reaction Time

The Pearson correlation coefficient (*r* = −0.556, *p* = 0.000) indicates a moderate negative correlation between emotional state and reaction time to safety hazards. The regression model passed the *F*-test (*p* = 0.000), and the emotional valence explains 30.9% of the workers’ reaction time to safety hazards (*R*^2^ = 0.309, SE = 0.832, *F* = 664.413).

The regression results are shown in Table 11, and the quantitative relationship between safety hazards reaction time and emotional valence of construction workers is shown in Equation (1).


(1) RT=−9.952E−5−0.556EV+ε

**Table 11:** Coefficients between reaction time and emotional valence.

	Unstandardized coefficients		Standardized coefficients	*t*	Sig.
	*B*	Standard error	Beta		
Constant(*β*_0_)	−9.952E-5	0.022		−0.005	0.996
Emotional valence(*β*_1_)	−0.556	0.022	−0.556	−25.776	0.000

Where

RT is safety hazards reaction time,
EV is emotional valence,
*ε* is error term, which indicates unexplained variability of the data.

**Figure 9:**
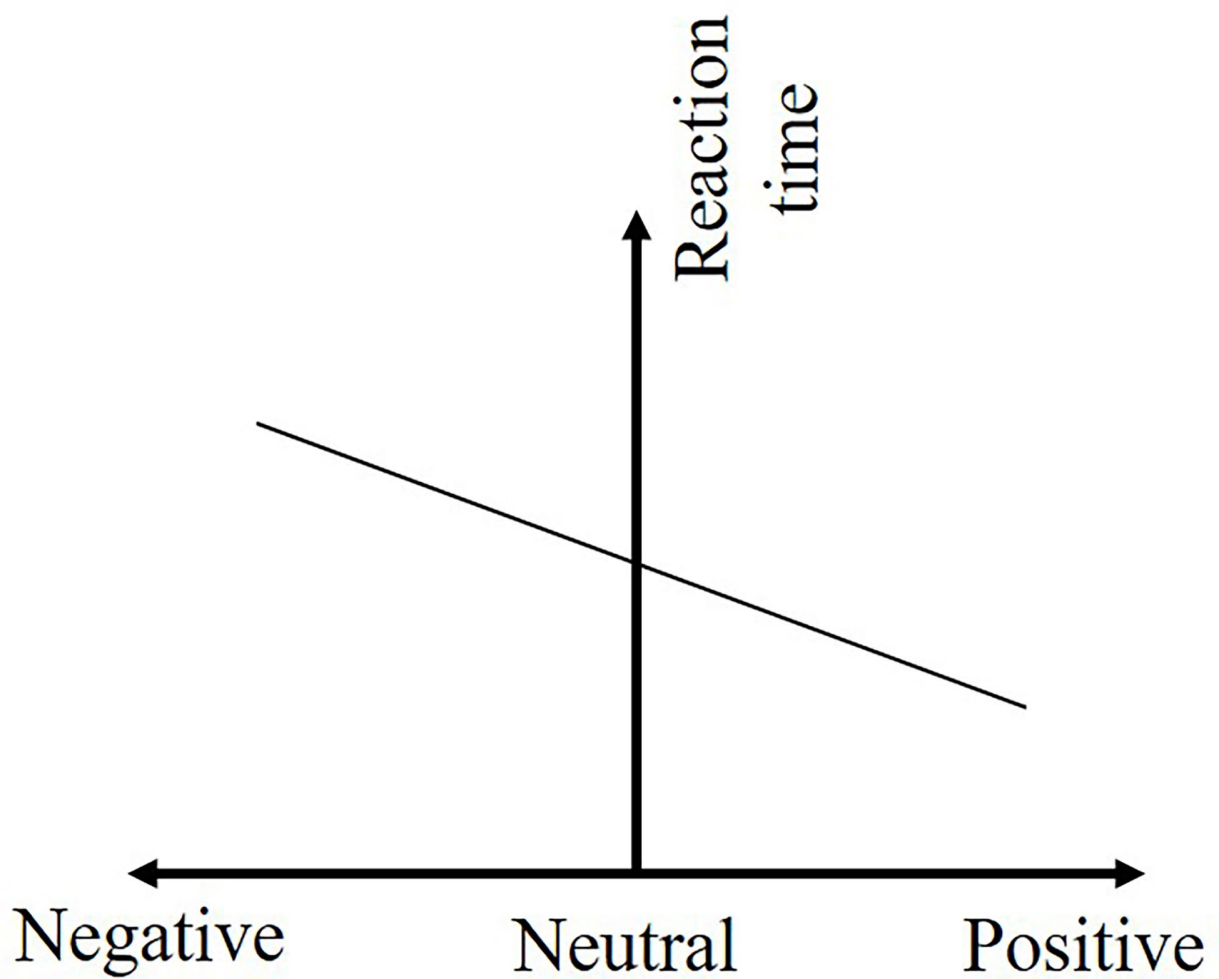
The effect of emotional valence on the reaction time to safety hazard.

The coefficient of emotional state and reaction time to safety hazards (*β_1_* = −0.556) is less than zero, indicating that the reaction time to safety hazards decreases as the emotional state increases, as shown in Figure 9. Either in positive or negative emotional state, the reaction time to safety hazards increased by 0.556 units for each unit decrease in the construction worker’s emotional valence. The reaction time to safety hazards decreases continuously as the emotional state of construction workers change from extreme negative to extreme positive.

#### Emotional Valence and Identification Accuracy

Descriptive statistics found that the identification accuracy of safety hazards was higher for construction workers in neutral emotions than in both positive and negative emotions. The Pearson correlation coefficient shows a moderate negative correlation between positive emotion and identification accuracy of safety hazards (r = −0.526, *p* = 0.000), while negative emotion is negatively correlated with identification accuracy of safety hazards (*r* = −0.356, *p* = 0.000).

The correlation analysis shows that the relationship between the emotional state and the identification accuracy of safety hazards is an inverted U-shape, so curvilinear regression model is established to show a quadratic expression. The regression model passed the F-test (*p* = 0.000) and the emotional valence explains 21.3% of the workers’ identification accuracy of safety hazards (*R*^2^ = 0.213, SE = 0.834, *F* = 201.79). The regression results are shown in Table 12, and quantitative relationship between identification accuracy of safety hazards and emotional valence of construction workers is shown in Equation (2).


(2) $$\mathrm{IA}=0.51+0.021\mathrm{EV}-0.443{\mathrm{EV}}^{2}+\varepsilon$$

**Table 12:** Coefficients between identification accuracy and emotional valence.

	Unstandardized coefficients		Standardized coefficients	*t*	Sig.
	*B*	Standard error	Beta		
Constant( $\alpha_{0}$ )	0.51	0.031		16.380	0.000
Emotional state( $\alpha_{1}$ )	0.021	0.022	0.023	0.981	0.327
Emotional state( $\alpha_{2}$ )	−0.443	0.022	−0.459	19.769	0.000

Where

IA is identification accuracy of safety hazards,
EV is construction worker’s emotional valence,
ε is error term, which indicates unexplained variability of the data.

**Figure 10:**
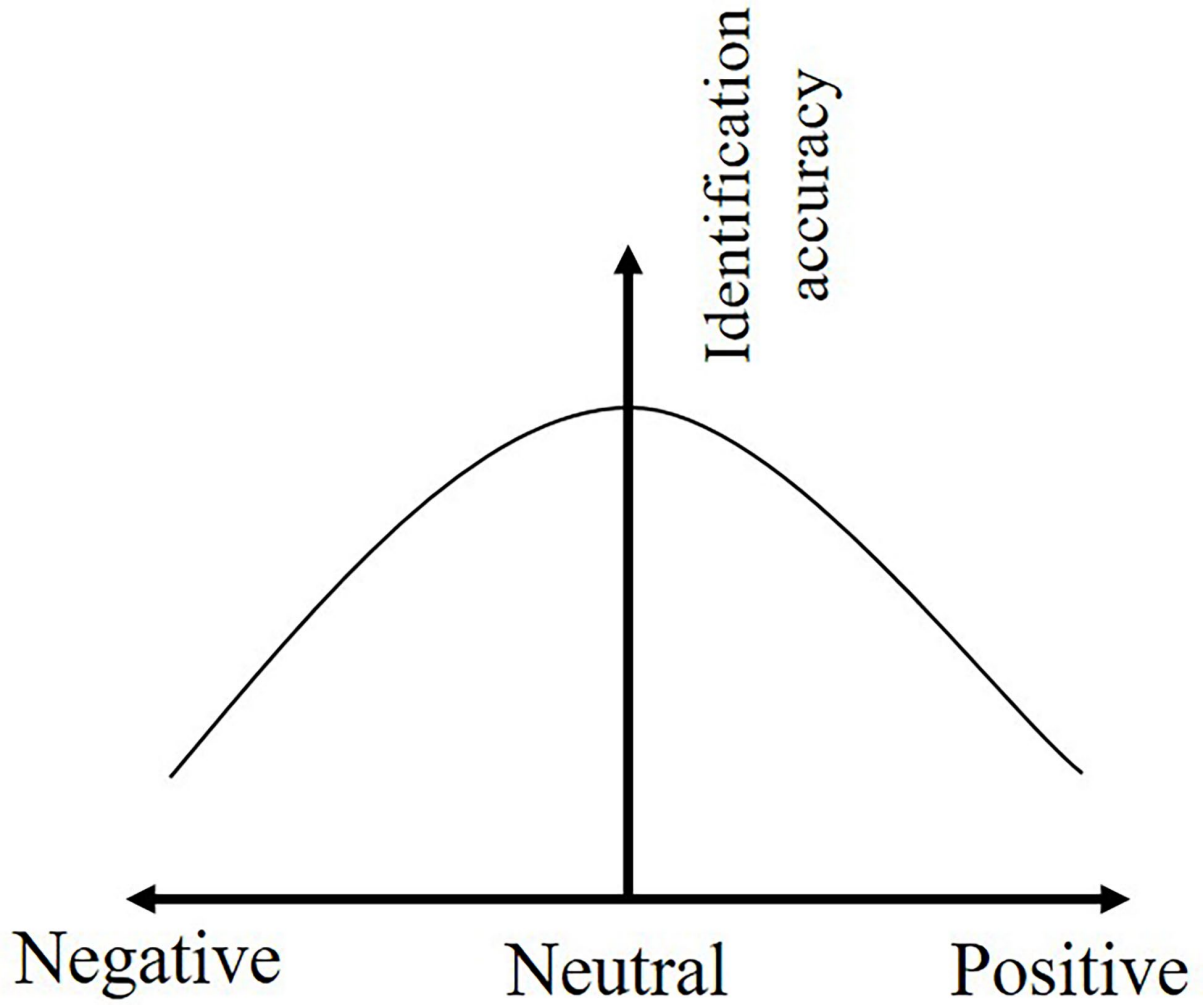
The effect of emotional valence on the identification accuracy.

The coefficient of the quadratic term ( $\alpha_{2}$ = − 0.443) between the emotional state and the accuracy of safety hazards identification is negative, indicating that the accuracy of safety hazards identification increases and then decreases as the emotional valence increases, reaching the highest point when the emotional state of workers is in neutral emotion as shown in Figure 10. Specifically, when the construction workers are in positive emotional state, the identification accuracy of safety hazards decreases by 1.350 units for each unit increase in emotional state. While the construction workers are in a negative emotional state, the identification accuracy of safety hazards decreases by 1.308 units for each unit decrease in their emotional states.

### Emotional Valence and Perception Level of Safety Hazards

The correlations between the positive emotion, negative emotion and the perception level of safety hazards were explored, respectively, by Pearson correlation coefficient. The results indicate a low negative correlation between positive emotion and perception level of safety hazards (*r* = −0.256, *p* = 0.000), while negative emotion is moderately negative correlated with the perception level of safety hazards (*r* = −0.520, *p* = 0.000).

The correlation analysis shows that the relationship between the emotional state and perception level of safety hazards is an inverted U-shape, so curvilinear regression is used to establish a quadratic expression for regression analysis. The regression model passed the *F*-test (*p* = 0.000), and the emotional valence explains 35.7% of the workers’ perception level of safety hazards (*R*^2^ = 0.357, SE = 0.802, *F* = 365.999). The regression results are shown in Table 13, and quantitative relationship between perception level of safety hazards and emotional valence of construction workers is shown in Equation (3).


(3) $$\mathrm{PL}=0.683-0.079\mathrm{EV}-0.617{\mathrm{EV}}^{2}+\varepsilon$$

**Table 13:** Coefficients between perception level of safety hazard and emotional valence.

	Unstandardized coefficients		Standardized coefficients	*t*	Sig.
	*B*	Standard error	Beta		
Constant( $\gamma_{0}$ )	0.683	0.034		20.354	0.000
Emotional state( $\gamma_{1}$ )	−0.079	0.021	−0.084	−3.747	0.000
Emotional state( $\gamma_{2}$ )	−0.617	0.023	−0.604	−27.055	0.000

Where

*PL* is perception level of safety hazards,
EV is construction worker’s emotional valence,
ε is error term, which indicates unexplained variability of the data.

**Figure 11:**
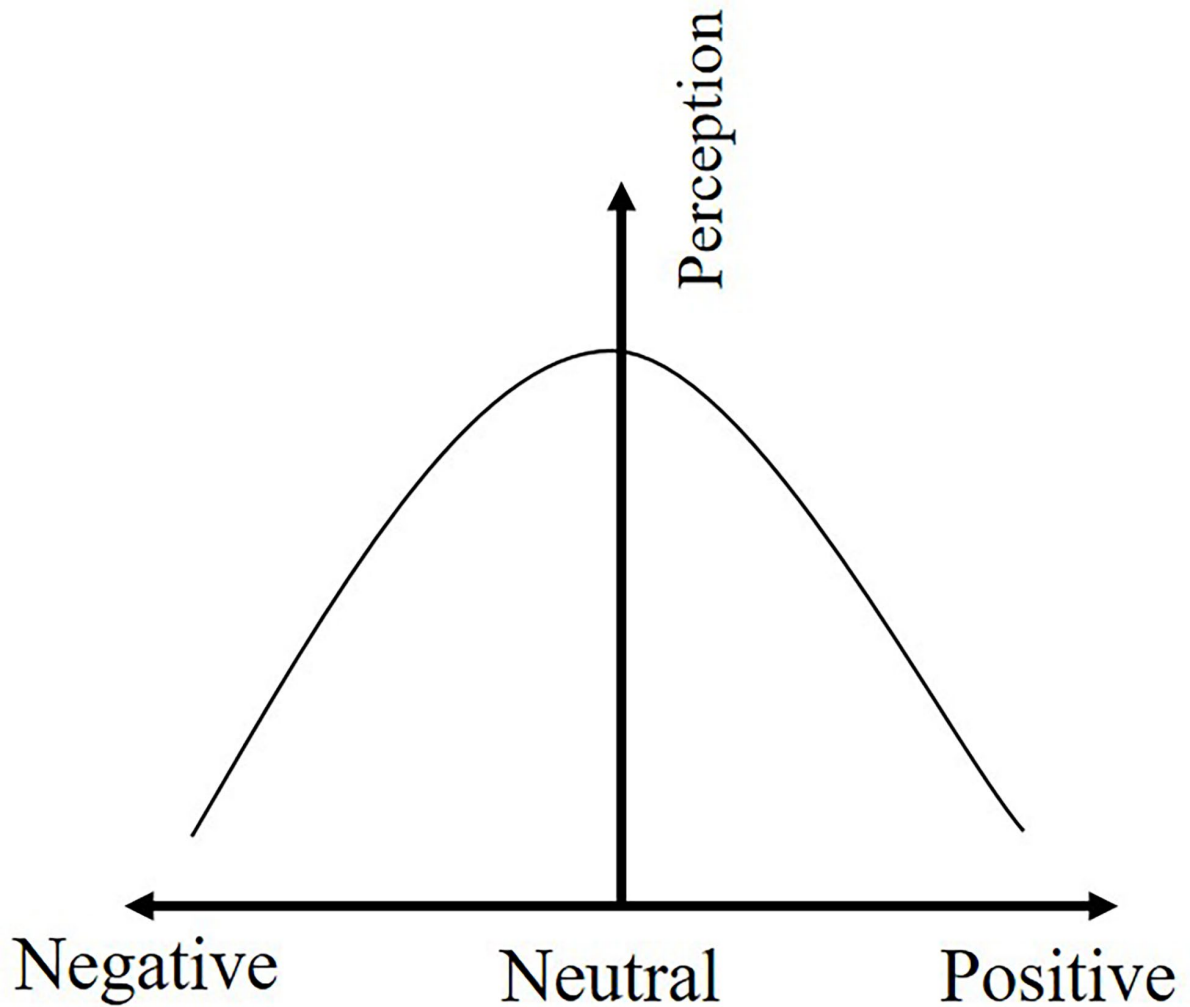
The effect of emotional valence on the perception level of safety hazard.

The coefficient of the quadratic term( $\gamma_{2}$ = − 0.617) between the emotional state and the perception level of safety hazards is negative, indicating that the perception level of safety hazards increases and then decreases as the emotional valence increases, reaching the highest point when the emotional state of workers is in neutral emotion as shown in Figure 11. Specifically, when construction workers are in positive emotion, the perception level of safety hazards decreases by 1.93 units for every each increase in their emotional state. When construction workers are in negative emotion, the perception level of safety hazards decreases by 1.772 units for each unit decrease in their emotional state. Therefore, the perception level of safety hazards of construction workers in negative and positive emotions is lower than that in neutral emotions. As construction workers’ emotions change from extremely negative to extremely positive, the perception level of safety hazards changes from low to high and then lower.

### The Safest Emotion

There was a negative correlation between reaction time to safety hazards and emotional valence, while the accuracy of safety hazards identification and the perception level of safety hazards had an inverted “U” shape relationship with emotional valence. When workers are under positive emotional valence, the findings are consistent with the affective generalization theory (Johnson and Tversky, 1983), where positive emotion drive construction workers to make optimistic judgements about the construction environment, and therefore lower levels of perception of safety hazards. When under the negative emotional valence, the findings are more in line with the Mood Maintenance Hypothesis (Isen and Patrick, 1983), where construction workers tend to take more risky and aggressive decisions in order to escape from their current negative emotional state, thus underestimating the risks of the environment and lowering the level of safety hazards perceived by workers. In addition, the findings of this study are support the Affective states as information hypothesis proposed by Schwarz and Clore (1981). This theory suggests that emotions simplify people’s risk decision-making process, which people judge things based on their feelings rather than their features, and that emotions can lead to overestimation of events of the same valence. This finding is related to information acquisition and cognitive processes, and subsequent research could be further explored from this perspective. Workers should avoid overexcited emotional states, for each unit increase in emotional valence, the reaction time to safety hazards reduced by 0.556 units. Meanwhile the identification accuracy of safety hazards reduced by 1.35 units, and the level of safety hazards perception reduced by 1.93 units when the emotional valence shift by one unit from neutral emotional state. Workers with high emotional valence have a more relaxed and pleasurable state, with increased reaction speed but reduced ability to judge and perceive safety hazards due to inattentiveness. Construction workers also need to avoid negative emotions such as excessive sadness and grief, for each unit decrease in emotional state, the feedback time for safety hazards recognition increases by 0.556 units. Meanwhile the identification accuracy of safety hazards decreases by 1.308 units and the perception level of safety hazards decreases by 1.772 units. When workers are immersed in a state of loss and frustration, the attention allocated to safety hazards identification decreases, prolonging their own judgment time, with a concomitant decrease in the accuracy of safety hazards identification and the level of safety hazards perception. Therefore, neutral emotions are the safest emotions.

## Conclusion

Behavioral experiment revealed that the support vector machine (SVM) algorithm was effective in classifying galvanic skin response signals to identify emotional states. The reaction time to recognition ability of safety hazards of construction workers under negative emotion is longer than neutral and positive emotions, and the identification accuracy of safety hazards and the perception level of safety hazards are lower, so the general recognition ability of safety hazards of construction workers under negative emotion is poorer. The reaction time to safety hazards identification is shorter for construction workers in positive emotions, but the accuracy of safety hazards identification and the level of safety hazards perception are lower, and the accuracy of safety hazards identification and the level of safety hazards perception are higher for construction workers in neutral emotions than in negative and positive emotions. For construction workers with more than 20 years of experience, work experience can effectively reduce the impact of emotional fluctuations on the accuracy of safety hazards evaluation. Emotion predicted the recognition ability of safety hazards of construction workers, with a moderate negative correlation between reaction time to safety hazards and emotional valence, and a low relationship between accuracy of safety hazards identification and perception level of safety hazards and emotional valence shaped an inverted “U.” Compared to positive emotions and negative emotions, construction workers in neutral emotions have the highest level of accuracy of safety hazards identification and perception of safety hazards, making neutral emotions deemed to be the safest emotion.

The complexity and dynamics of the construction site require workers to identify the safety hazards present on the site timely and accurately, and keeping their emotional state stable is beneficial to improving construction workers’ ability to identify safety hazards and keep themselves safe. Currently, China’s construction workers are generally poorly educated, lack continuous psychological training and have weak emotional control, while safety training in construction companies tend to focus on the operational specifications, unsafe behaviors, the requirements for wearing safety gear and the main prohibitions of safe production, and rarely include emotional management and requirements in safety education and training. Construction companies should pay more attention to the emotional health of construction workers and keep their emotional state stable through psychological training, to improve workers’ awareness of their emotion and emergency handling ability, therefore to reduce the probability of safety accidents and improve the safety management of construction sites.

There are some limitations to the present study which may be relevant for future research. First, the study used galvanic skin response to monitor emotions, while scientific and technological advances have led to increasingly sophisticated techniques for monitoring physiological signals. Some studies focus on collecting EEG signals through EEG devices (Takehara et al., 2020; Long et al., 2021), exploring brain activity in different emotional states. Eye-tracking devices were used to collect construction workers’ eye-movement signals, and analyses how eye-movement signals reflect the emotions of construction workers (Soleymani et al., 2012). Future research could investigate the impact of emotions on an individual’s physiological signals, as well as cognitive abilities, in a multidimensional approach through the applications of novel devices. Second, the participants in this study were mainly construction workers, and this paper explored the effect of work experience on construction workers’ emotions. Future research could further focus on the variability within groups of construction workers, such as different personality traits (Sugi et al., 2020; Maier et al., 2021), different populations (Gutiérrez-Cobo et al., 2017) or gender (Lischke et al., 2020), to explore differences in the influence of emotions between groups of construction workers. At last, the study categorized emotions into the three most basic types, negative, neutral and positive by emotional valence. In fact, there are more varieties of emotions and even research paradigms, research on different negative emotions has gained extensive attention (Pittig et al., 2014; Topolinski and Strack, 2015), and subsequent research can be conducted from these perspectives and be studied in more detail.

## Data Availability Statement

The raw data supporting the conclusions of this article will be made available by the authors, without undue reservation.

## Ethics Statement

The studies involving human participants were reviewed and approved by the Ethics Committee of Shanghai University.

## Author Contributions

DC, AY, and HS contributed to conceptualization, writing— review and editing, formal analysis, methodology, and original draft. HS and DC contributed to investigation. DC and YZ contributed to supervision. All authors contributed to the article and approved the submitted version.

## Funding

This study was funded by the National Natural Science Foundation of China (grant no. 71901139) and Science and Technology Commission of Shanghai Municipality (grant nos. 19DZ1204203 and 21692195100).

## Conflict of Interest

YZ is employed by Shanghai Road & Bridge (Group) Co., Ltd.

The remaining authors declare that the research was conducted in the absence of any commercial or financial relationships that could be construed as a potential conflict of interest.

## Publisher’s Note

All claims expressed in this article are solely those of the authors and do not necessarily represent those of their affiliated organizations, or those of the publisher, the editors and the reviewers. Any product that may be evaluated in this article, or claim that may be made by its manufacturer, is not guaranteed or endorsed by the publisher.
